# Air-Cladding Blue Laser Diodes

**DOI:** 10.1021/acsami.6c01600

**Published:** 2026-06-20

**Authors:** Marta Sawicka, Mateusz Hajdel, Oliwia Gołyga, Henryk Turski, Mikołaj Chlipała, Anna Feduniewicz, Szymon Stańczyk, Czesław Skierbiszewski, Cedric Corley-Wiciak, Carsten Richter, Grzegorz Muziol

**Affiliations:** † 243577 Institute of High Pressure Physics Polish Academy of Sciences, Sokołowska 29/37, Warsaw 01-142, Poland; ‡ 55553 European Synchrotron Radiation Facility, X-ray Imaging and Microscopy Group, 71 Avenue des Martyrs, CS 40220, Grenoble 38043, France; § 28399 Leibniz -Institut für Kristallzüchtung, Max-Born-Straße 2, Berlin D-12489, Germany

**Keywords:** laser diodes, nitrides, electrochemical etching, membrane devices, scanning X-ray diffraction microscopy

## Abstract

Low refractive index contrast in long-wavelength nitride laser diodes (LDs) limits optical confinement, motivating new architectural approaches. Here, we report the first electrically driven edge-emitting LDs featuring top and bottom air-claddings. To enable the top air cladding, the architecture employs a tunnel junction, which converts the current flow from holes to electrons. This allows for low series resistance lateral current flow and placement of the metal contact on the side of the LD mesa. The bottom air-cladding is realized postepitaxy through lateral electrochemical etching (ECE) of a highly doped InGaN:Ge sacrificial layer. Depending on the geometry of the openings for electrolyte access, wing-like and membrane LD devices are obtained. Very high backside smoothness of the membrane has been achieved thanks to an abrupt doping profile and excellent selectivity in material removal by ECE. Synchrotron-based scanning X-ray diffraction microscopy shows that laser membranes exhibit slight elastic relaxation, which results in bending of the LDs by a few nanometers over a distance of a dozen microns. LDs with dual air-claddings operated in pulse mode at a wavelength of λ = 456 nm with a slope efficiency of 0.4 W/A, similar to their reference counterparts without under-etching. This architecture is expected to provide greater benefits of refractive index engineering for longer wavelength LDs, where high refractive index contrast is more challenging. Moreover, the work highlights ECE as an extremely effective method for device liftoff, enabling GaN substrate reuse and facilitating transfer and integration of LDs into advanced photonic platforms, including therapeutic applications.

## Graphical abstract

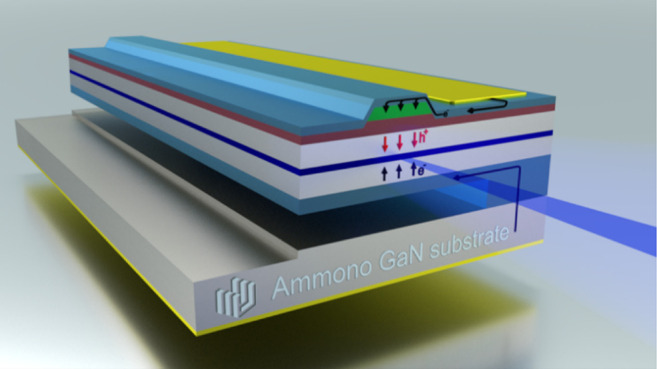

## Introduction

Electrochemical etching (ECE) has recently been integrated into the fabrication process of a wide range of III-nitride structures and devices because it allows for the introduction of porosity in a selected layer or its complete removal, leaving an air gap.^1–3^ Porous GaN emerges as a particularly interesting material in the nitride family because of its tunable refractive index, large surface area, and reduced mechanical stability. Therefore, the applications of porous GaN layers range from highly refractive distributed Bragg reflectors (DBRs),^4^ vertical surface-emitting lasers (VCSELs),^5^ and resonant cavity light-emitting diodes (RC LEDs)^6^ to the utilization of porous GaN for enhanced water splitting^7^ or as a semiflexible underlayer enabling strain compliance.^8,9^ Due to the fact that the refractive index of porous GaN can be tailored in a very broad range, essentially down to *n*_*air*_ = 1, it was proposed as an alternative material to AlGaN claddings, and enabling an effective increase of the optical confinement factor in optically pumped blue lasers.^10^ Following that demonstration, electrically pumped edge-emitting laser diodes (LDs) with porous bottom cladding, emitting in blue and green, have been reported.^11–14^ Replacing AlGaN claddings with a material that is both lattice-matched to GaN and offers a higher refractive index contrast to GaN is especially beneficial for long- and short-wavelength LDs. It solves the key challenges related to strain engineering of thick claddings required for proper light confinement.^15,16^

Importantly, ECE of n-type GaN not only enables the formation of a porous structure but can also be used for the full removal of a selected layer. The conditions for complete etching of a sacrificial layer depend on the doping level, etching voltage, electrolyte type, and carrier concentration,^17–20^ which are the same parameters that play a role in controlling porosity and pore morphology. Importantly, the full removal of the sacrificial layer with the aid of lateral ECE or photoassisted ECE is vital for several new technologies. First, efficient structure liftoff^21,22^ has been utilized for microtransfer printing of blue LEDs^23,24^ or precise control of cavity length in ultraviolet-C VCSELs.^25^ Future integration of transfer-printed nitride LDs with photonic integrated circuits (PICs) has the potential to enable significant miniaturization while facilitating optical signal processing, sensing, and novel consumer electronics applications. Second, one can imagine the application of the ECE technique for aperture diameter definition in electrically pumped VCSELs. Third, the formation of microchannels within the GaN substrate allows for microfluidic cooling close to the heat source, forming an efficient heat dissipation system, as has been recently demonstrated for GaN on Si electronics.^26^ Fourthly, the miniaturization enabled by ECE allows for the application of LDs in optogenetics and therapeutic devices, which are now limited to LEDs.^27–29^ Lastly, if device liftoff is realized on the whole wafer, GaN substrate reuse becomes possible, which significantly reduces the expenses spent for high-quality substrates.^29^

This work demonstrates a new architecture of LDs with air claddings on both sides of the active region. The top air-cladding is provided by the metal-free laser ridge enabled by a tunnel junction (TJ).^30,31^ The bottom air cladding is achieved by the selective removal of a sacrificial, highly n-type doped InGaN:Ge layer through ECE. We demonstrate pulse-mode operation of such blue air-cladding LDs. The lasing wavelength is 456 nm, and the slope efficiency reaches 0.4 W/A, with a comparable or better threshold current density than that of non-etched reference LD structures from the same wafer. The created floating resonators have an atomically smooth backside surface and show only a few nanometers of bending at a distance of a dozen micrometers. Finally, we also compare the benefits of such an air-cladding architecture for long-wavelength emitters, showing a greater enhancement in the optical confinement factor for green and red emitters as compared to blue.

### Fabrication of Air-Gap in Laser Diode

The epitaxial structure was grown by plasma-assisted molecular beam epitaxy (PAMBE) on a (0001) Ammono-GaN substrate in a Veeco Gen20A reactor. The layer sequence is presented in Figure 1a. Full details about the layer thickness and doping are given in the Methods section.

**Figure 1:**
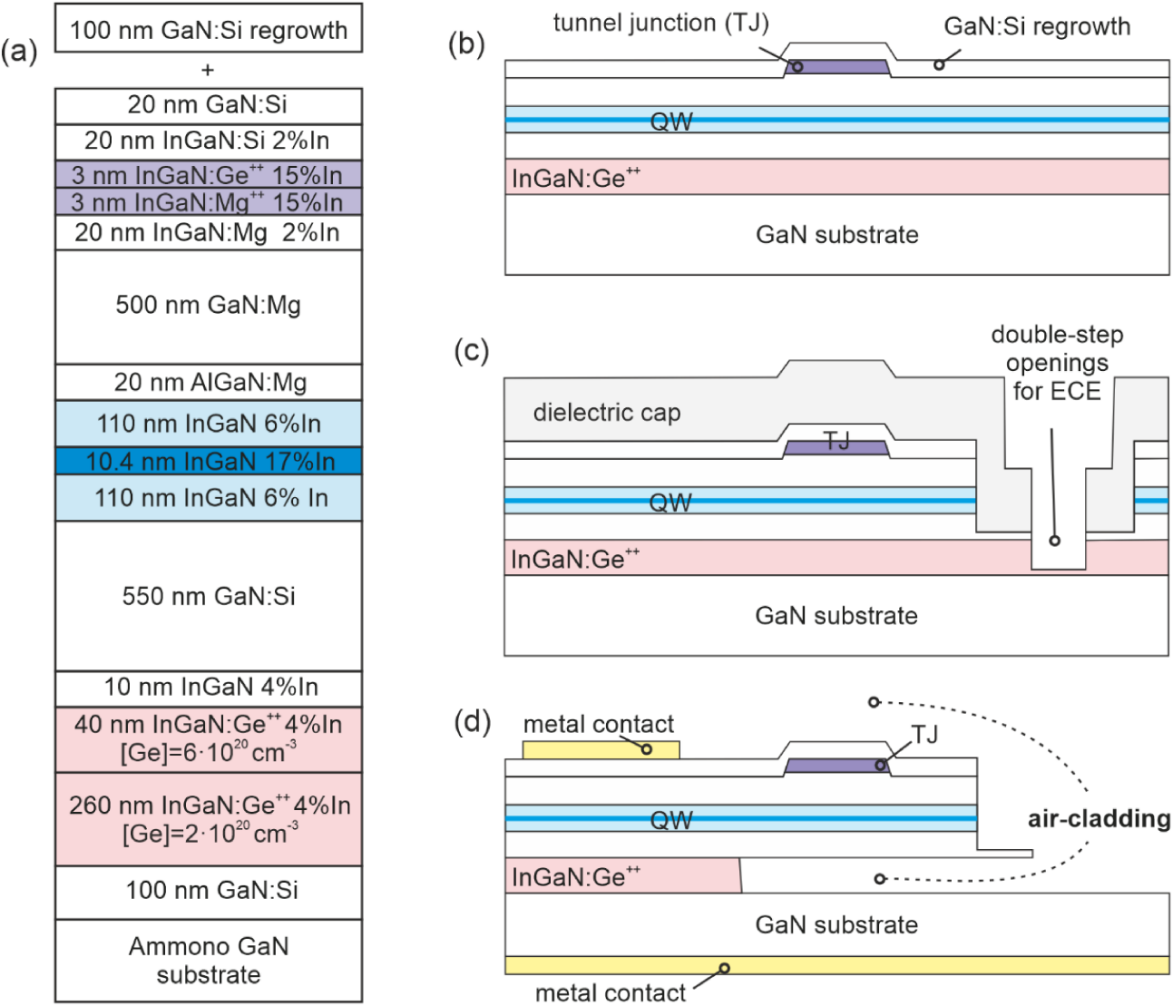
(a) Schematic of the epitaxial structure of the air-clad LD. (b–d) Illustration of the LD processing steps: (b) epitaxy, mesa formation, and regrowth; (c) structure with a protective step, capped with a dielectric cap, and openings for ECE access to the sacrificial layer; (d) schematics of an air-cladding LD after processing.

The device processing sequence is schematically illustrated in Figure 1b–d. It was adopted from our previous work on the porous-cladding LDs,^13^ and it includes (i) the epitaxy of a laser structure with a top TJ, mesa formation, and regrowth of the GaN:Si layer, (ii) protecting the structure with a dielectric cap and forming openings for ECE, and (iii) ECE and contact deposition. A GaN wafer with an LD epi-structure was cleaved into two parts: the bigger piece underwent processing, while the smaller one was used to test ECE conditions and was examined at the European Synchrotron Research Facility (ESRF). Processing details are given in the Methods.

Two geometries of the openings for ECE were designed: long grooves and square-shaped windows, both including double-step protection geometry to prevent electrolyte access to the active region and the TJ. Long grooves parallel to laser ridges resulted in ***winglike lasers***, while the window-type openings resulted in ***membrane*** ***lasers***. Optical microscope images showing the formation of bottom air cladding in both types of openings—window-type and groove openings for ECE—are presented in Figure 2a,b. Figure 2c shows the optical microscope image of a larger part of the crystal, in which three kinds of devices are visible: no-etch, winglike, and membrane air-clad LDs.

**Figure 2:**
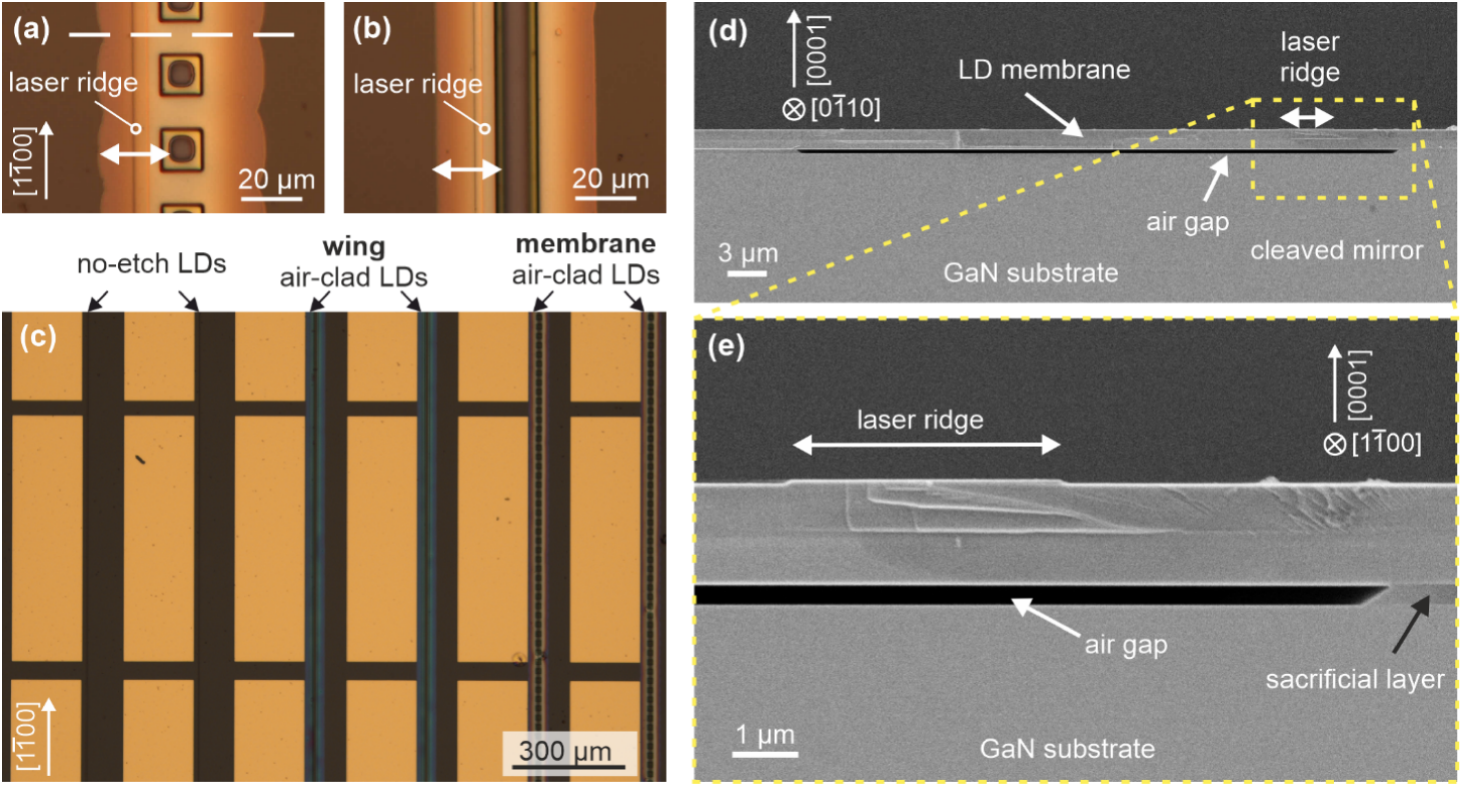
Optical microscope images showing (a) membrane LDs and (b) wing-like air-clad LDs. The position of the cleavage is schematically marked with a dashed line. (c) Processed wafer before device cleaving, with lasers having different claddings. Positions of the ridges are marked with arrows. (d) SEM image of the cleaved wafer presenting the laser membrane after sacrificial layer removal, viewed along the [01̅100] direction. (e) Magnified area of the laser ridge and the air-gap beneath.

The removal of the sacrificial layer was confirmed by scanning electron microscopy (SEM) on single laser chips and cleaved laser bars. Figure 2d,e present the SEM cross-sectional views of the laser membrane. The cleavage plane intersects the bridge between window openings for ECE, and its position is indicated in Figure 2a by a white dashed line. The SEM image is oriented along the [01̅100] direction. Figure 3a and b show SEM images of the membrane and wing-like laser chips, respectively, acquired at an oblique viewing angle with the laser chips inclined by approximately 45°. The presented cross-sectional observations consistently confirm that the sacrificial layer has been effectively removed, resulting in the formation of an air gap beneath the ridge.

**Figure 3:**
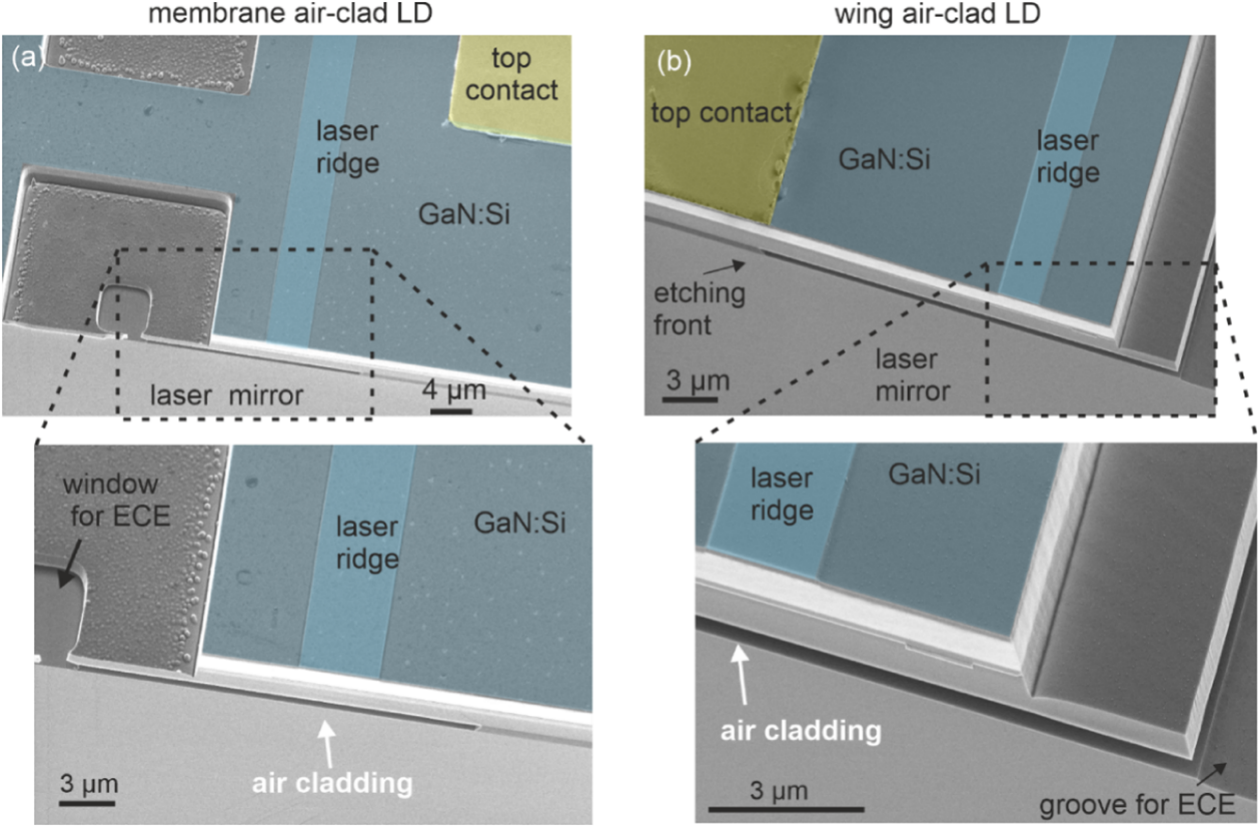
False-colored SEM images of the fabricated air-cladding LDs in (a) membrane- and (b) wing-like geometries, viewed at an arbitrary angle, showing the laser ridge and metal contact. Magnifications are presented for the air-cladding formed below the ridge. Openings for ECE (window and groove) are marked with black arrows.

### Air-Cladding Laser Structure Deformation Studies at Submicron Lateral Resolution

Despite the fact that SEM imaging of the laser structures showed no buckling or bending of the laser wings or membranes (cf. Figure 3), it is expected that the introduction of an air gap to the laser structure, as well as windows for ECE that partially remove in-plane geometric restrictions, impacts its strain state. However, due to the small surface area of the under-etch, the use of X-ray diffractometry and standard reciprocal space mapping (RSM) was not possible because it lacks the spatial resolution needed to verify lattice relaxation or deformation. Therefore, we employed synchrotron-based scanning X-ray diffraction microscopy (SXDM) at the hard X-ray nanoprobe beamline ID01/ESRF to image crystal lattice distortions on a submicron length scale.^32,33^ This nondestructive method is capable of mapping local lattice bending and strain in epitaxial layers with very high sensitivity, down to 10^–5^ and spatial resolution ≤50 nm.^34^

We examined the test-piece sample that had no metal contact deposited and an air-gap under-etched for a distance of 13.5 μm from the opening for ECE. Two geometries of the openings for ECE were measured – windows and grooves, resulting in membrane- and wing-type lasers, respectively.

For both geometries, we first compared the RSMs taken in the reference position outside of the air gap and compared them to the RSMs taken above the air gap (in the area of a membrane and wing—left and right of the ECE opening; see Figure S2 and S3 in the Supporting Information). In both cases, the GaN peaks obtained above the under-etched regions are shifted to higher momentum transfer *Q*_*z*_, corresponding to a smaller out-of-plane lattice parameter *c*, in comparison to the reference region. Note that *Q*_*z*_ and the lattice parameter c are related as *Q*_*z*_ = 2π × 6/*c*. Furthermore, the radial cuts of the RSMs *I*(*Q*_*z*_) show that the InGaN peak located at a lattice parameter of c ≈ 5.218 Å, corresponding to ≈4% indium, disappears, which is expected due to the etching of the sacrificial InGaN layers, as marked in pink in Figure 1 (d). The remaining InGaN peak is located at a lattice parameter of c ≈ 5.2317 Å, which corresponds to strained InGaN with an indium concentration of 5.8%; see Figure S4 and S5 in Supporting Information.

This shift of GaN peaks observed in under-etched regions is expressed to a different extent for the two different etching geometries: from 5.1855 Å to 5.1849 Å (ε_zz_ = −0.125 × 10^–3^) for the membrane structure and from 5.1856 Å to 5.1836 Å (ε_zz_ = −0.386 × 10^–3^) for the wing-like LD. We attribute this compressive out-of-plane strain as a response to the lateral elastic relaxation of the under-etched regions, enabled by the ECE opening and the under-etching, both of which remove geometric restrictions that maintain the pseudomorphic strain in the reference region.^35^ Based on a typical Poisson number for GaN of *ν*_13_ ≈ 0.2,^36^ which connects in-plane and out-of-plane strain by the equation

1 $$\varepsilon_{\mathrm{zz}}=\frac{\varepsilon_{\mathrm{xx}}+\varepsilon_{\mathrm{yy}}}{\frac{1}{v}-1}$$

and assuming relaxation takes place only toward the ECE opening (*ε*_yy_ = 0), this would imply a lateral elastic strain of the two GaN layers adjacent to the InGaN QW in the free-standing part of *ε*_xx_ = 0.50 × 10^–3^ for the membranes and *ε*_xx_ = 1.5 × 10^–3^ for the wings. Assuming no additional plastic relaxation is taking place, the same amount of strain would be elastically relaxed in the InGaN QW layers, resulting in a relative relaxation of 8% and 24% for membranes and wing-like structures, respectively. This assumption is supported by the observation that the InGaN (5.8% In) peaks also shift to higher *Q*_*z*_ in the radial cuts by a similar amount as the GaN peaks.

Second, we processed the RSMs for each point and extracted spatial maps of the integrated intensity of the 006 InGaN reflection and the angular deviation (tilt) of the (0006) lattice planes in terms of the angles φ (Phi) and θ (Theta), corresponding to a rotation of the lattice planes around the two in-plane crystallographic axes ⟨112̅0⟩ and ⟨11̅00⟩, commonly referred to as the *a*- and *m*-direction, respectively. Figure 4a,b shows the intensity maps for membrane and wing-like geometry, respectively, in which we can clearly distinguish the position of the 15 μm-wide windows and groove of low intensity (dark blue), since there is no InGaN left in these areas. The highest intensity (yellow) is observed in the unetched areas, as the signals from In_0.04_Ga_0.96_N sacrificial layer and In_0.06_Ga_0.94_N waveguide add up. Interestingly, the ridge placement is slightly visible, which is the only place where the InGaN TJ was left and contributes to the signal. Figure 4c,d and e,f show lattice tilt maps of the air-clad LDs around two axis ⟨112̅0⟩ and ⟨11̅00⟩, respectively, presenting the impact of elastic stress relaxation via the removal of elastic restrictions through the window/groove. The free-standing regions of the epitaxial layer stack may bend to relax the net elastic stress that adds up at each heterointerface and which would be zero if the layer thicknesses and lattice mismatch was symmetrical around the QW layers. We observe that the membranes bend downward, while the wing-like LDs bend upward. Wing-like lasers are bent only about the ⟨11̅00⟩ axis, while the membranes experience more complex bending behavior since there are free surfaces along both the m- and a-directions. Figure 5 presents the derived absolute displacement in the out-of-plane direction taken from the maps of the θ angle along the cross sections presented in Figure 4e-f. The magnitude of inclination in both cases is quite small, below 10 nm at the membrane/wing length of ∼ 11 μm.

**Figure 4:**
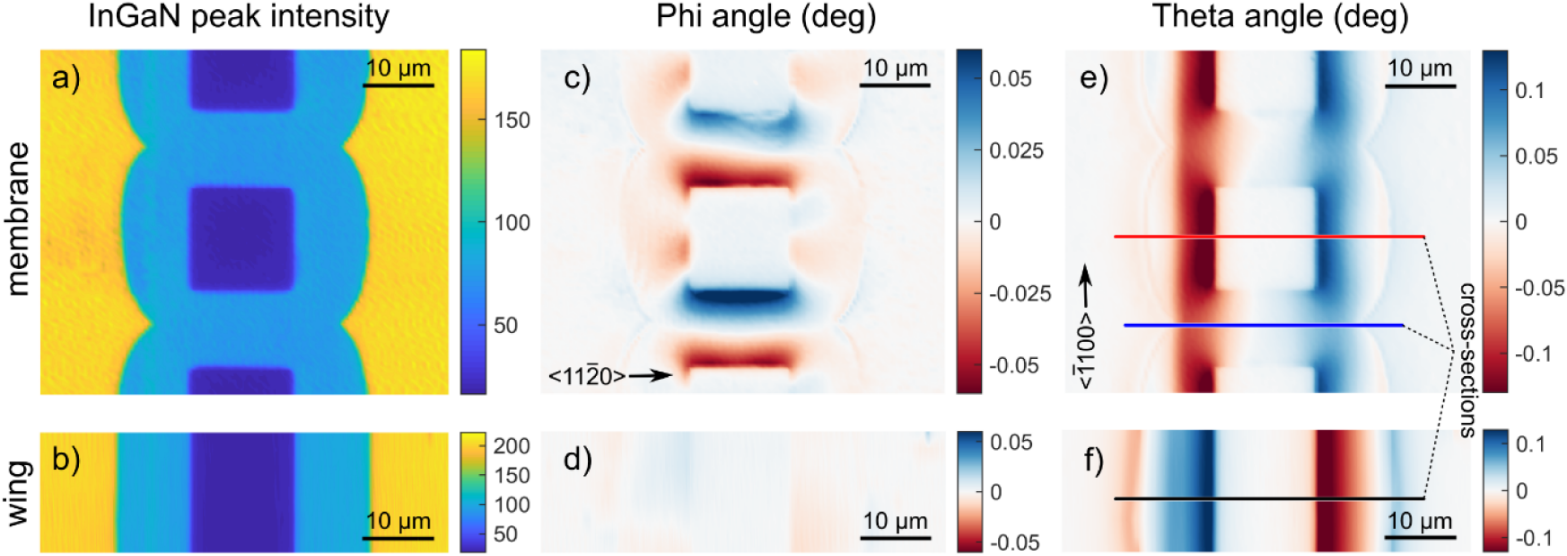
InGaN peak intensity map of (a) a membrane (window opening) and (b) a wing-like (groove) structure. Lattice tilt maps about the ⟨112̅0⟩ axis for (c) membrane and (d) wing-like structures, and about the ⟨11̅00⟩ axis for (e) membrane and (f) wing-like structures. The cross-sections used in Figure 5 are marked with solid lines in matching colors.

**Figure 5:**
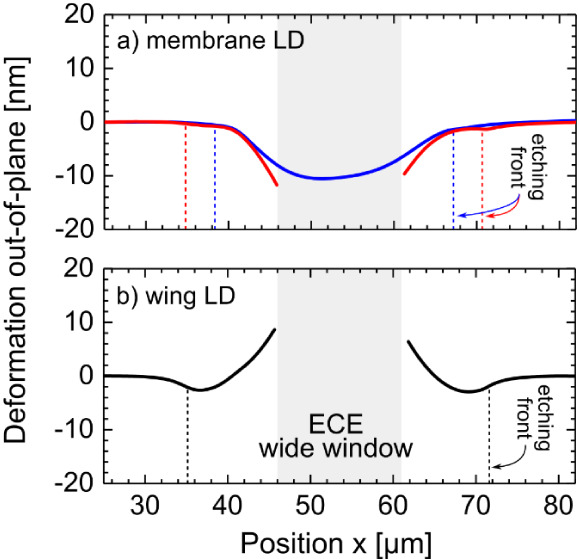
Deformation in the out-of-plane direction for (a) membrane and (b) wing-like LDs. For the membrane LD, two cross-sections are presented: at the center of the square-shaped windows (red solid line) and between the windows (blue solid line). The etching front position for the respective samples is marked with dashed lines.

### Characterization of Membrane Backside Surface after Sacrificial Layer Removal

In air-cladding LDs, one might expect scattering due to the roughness of the GaN/air interface. Especially, the interface of the bottom air-cladding needs to be considered as a possible source of scattering since it is formed by lateral ECE. The smoothness of the bottom surface of the air-clad LD was, therefore, investigated by atomic force microscopy (AFM). LDs were delaminated from the substrate using adhesive tape. No mechanical scratching was involved. Details of the preparation for the AFM measurements, together with additional SEM images of the backside of the membrane and the processed wafer from which it was taken, can be found in Figure S1 in the Supporting Information. Figure 6a presents the images of the backside of the air-clad LD (below the ridge) measured on a 5 × 5 μm^2^ area. The root-mean-square (rms) surface roughness is 0.66 nm, which is very similar to the value of 0.6 nm reported by Chlipała et al.^23^ This roughness is related to the slightly wavy surface morphology of the highly doped In_0.04_Ga_0.94_N:Ge layer. The roughness of the bottom air-cladding interface is small and should not introduce significant light scattering. Achieving such high surface smoothness is possible due to the abrupt doping profile when indium is used as a surfactant during the growth of Ge-doped InGaN layers.^37^ Figure 6b presents the surface of the Ammono-GaN substrate with an MBE-grown 100 nm GaN:Si after LD detachment (cf. the full epitaxial structure in Figure 1a, with an rms roughness of 0.32 nm. Parallel atomic steps can be distinguished with a step distance corresponding to the initial GaN substrate miscut of 0.5° toward the m-direction. After such a device liftoff process, the substrate could be reused.

**Figure 6:**
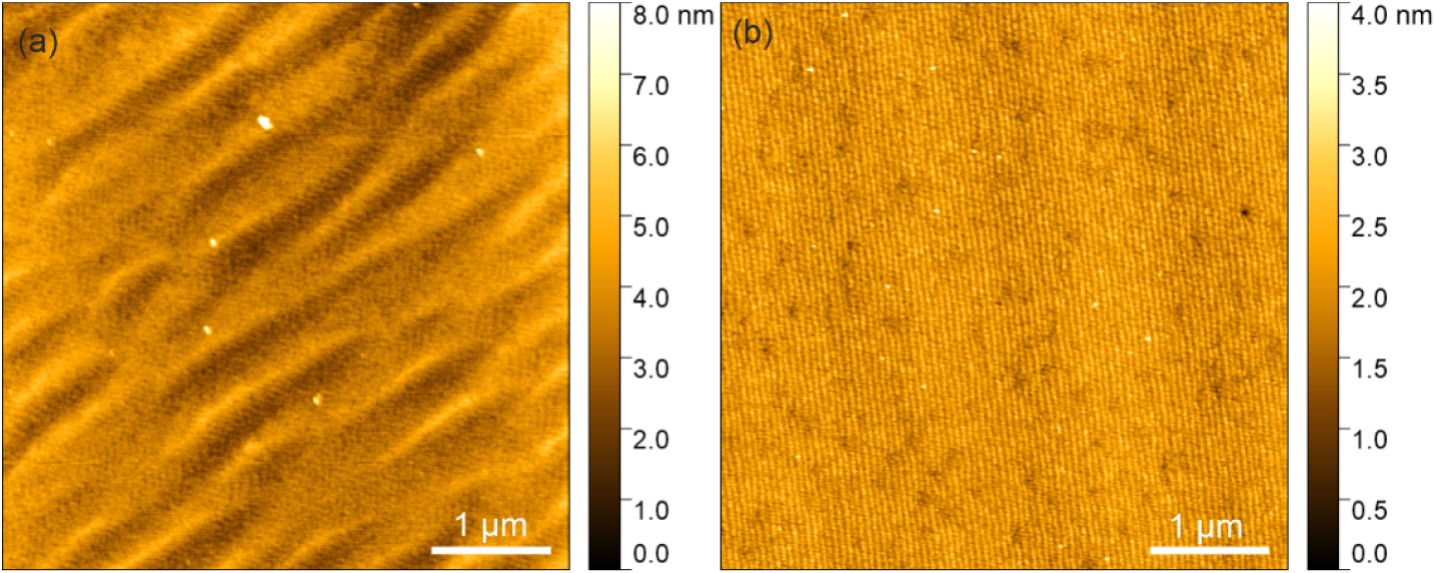
AFM images taken after laser membrane detachment: (a) on the backside of the LD membrane and (b) on the GaN substrate after sacrificial layer removal and detachment of the LD structure.

### Air-Cladding LDs: Electrical and Optical Characterization

The LD structure was processed to fabricate ridge-waveguide lasers with a resonator length of 1000 μm. The device mesa width was 5 μm. Laser mirrors were cleaved and left uncoated. Optical power was measured from a single laser facet. Light-current (L-I) characteristics and high-resolution spectra for the representative devices from the same wafer are presented in Figure 7a. The devices operated under pulse mode with a duty cycle of 0.05% and a pulse duration of 200 ns, with the temperature set to 20 °C at the Peltier cooler. No damage to the LD chips was observed after the electrical characterization of the devices in pulsed mode, despite the fact that heat dissipation occurs only through the thin connections with the substrate. The threshold current of the membrane air-clad LD is around 500 mA, while for the non-etched reference LD, it is 660 mA. That corresponds to a threshold current density, *j*_*th*_, of 10.0 and 13.2 kA/cm,^2^ respectively. The threshold current for the wing-type air-clad LD was 880 mA (*j_th_* = 17.6 kA/cm^2^). Thus, j_th_ is comparable for both types of air-clad LDs. The slope efficiencies of membrane, wing-like air-clad LDs, and non-etched LDs were 0.240, 0.414, and 0.179 W/A, respectively. The slope efficiency of an LD from a single uncoated facet is given by Coldren et al.^38^ as

2 $$\frac{dP}{dI}=\frac{1}{2}\eta_{i}\frac{\alpha_{m}}{\alpha_{m}+\alpha_{i}}\frac{h\nu }{e}$$

where $\eta_{i}$ *η*_*i*_ is the injection efficiency, *α*_*m*_ and *α*_*i*_ are the mirror and internal losses, respectively, *h* is Planck constant, *ν* is the light frequency, and *e* is the elementary charge. For simplicity, let us assume that the *η*_*i*_ is constant and equal to 0.5 for the studied lasers. For a resonator length of 1000 μm and a facet reflectivity of 0.18, the mirror losses are *α*_*m*_ = 17.2 cm^–1^. Assuming identical mirror losses for all the lasers, the internal losses *α*_*i*_ would be equal to 32, 11, and 49 cm^–1^ for membrane-, wing-, and non-etched LDs, respectively. The largest *α*_*i*_ in the case of the non-etched LD might be assigned to absorption by free carriers in the heavily doped InGaN:Ge sacrificial layer, which is present in the non-etched LD and there might be a nonzero overlap of the optical mode in this layer. However, the large difference between the two types of air-cladding layers is unexpected. Interestingly, it might be due to scattering on the wavy structure of the membrane-like LD, which was measured using SXDM and shown in Figure 4 as a change in the Phi angle. Such a wavy structure was not observed in the case of the wing-like LD. It needs to be noted that there are several sources of error in these considerations, which might significantly influence these conclusions: (i) standard cleaving of the facets in air-cladding LDs might lead to fractures on the facet of the LD and a different facet reflectivity, and (ii) the assumption of *η*_*i*_ 0.5 might be incorrect, since simulations show that *η*_*i*_ depends on current density,^39^ and the studied lasers operate at different threshold current densities (10.0, 17.6, and 13.2 kA/cm^2^, for membrane-, wing-, and non-etched LDs, respectively). Further studies are necessary to confirm the presented conclusions.

**Figure 7:**
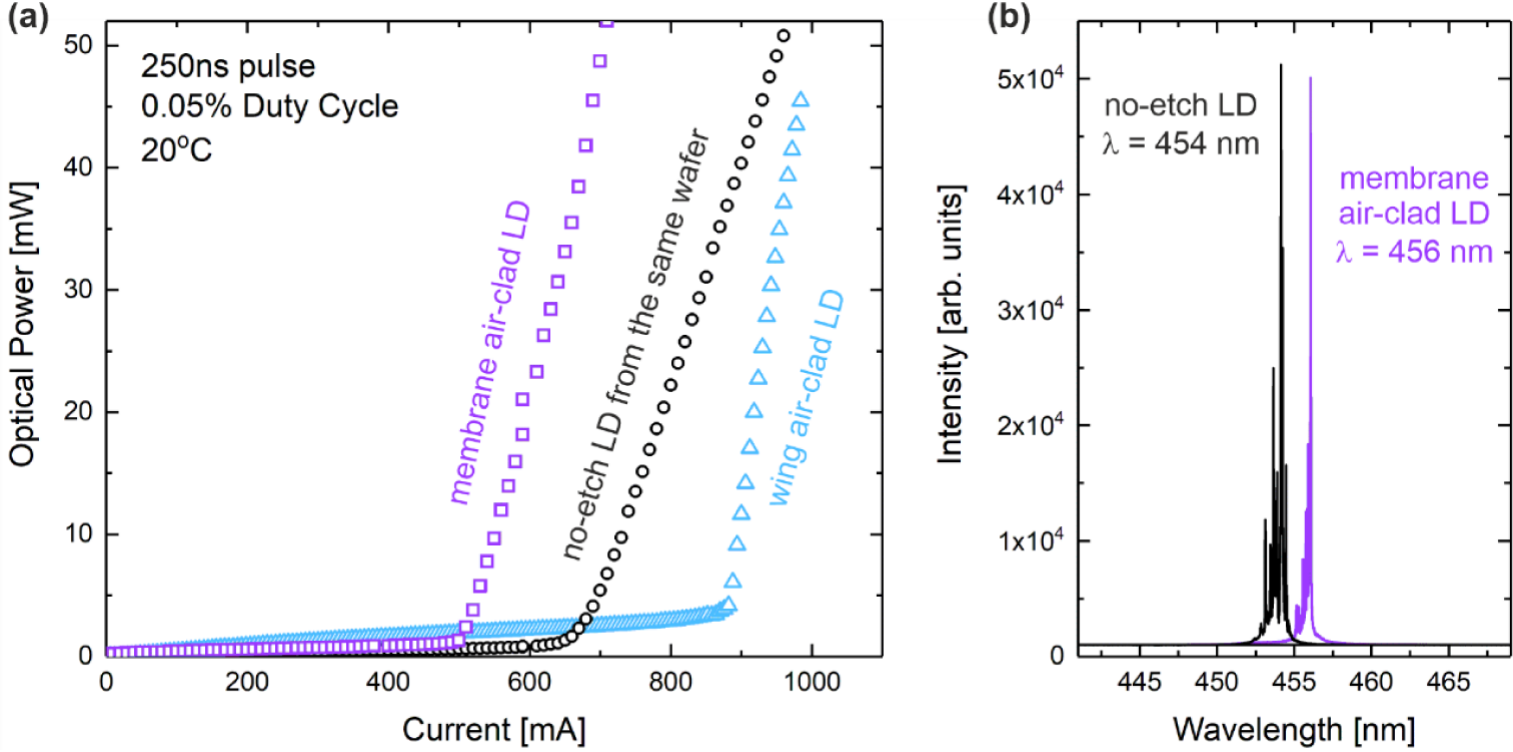
(a) Light–current (L–I) characteristics of membrane and wing-like air-cladding LDs, and non-etched LDs from the same wafer, operated in pulse mode. (b) High-resolution spectra measured at 510 and 800 mA, respectively.

Note that, in the case of porous bottom cladding blue LDs reported earlier, their slope efficiencies were significantly—15 times—smaller than their reference counterparts, i.e., 0.046 W/A and 0.692 W/A, respectively.^13^ The cause of the smaller slope efficiency is increased internal optical losses, most probably due to scattering on nanopores. The fact that we do not observe a decrease in slope efficiency in air-cladding LDs is an indication that the smooth backside of the air-clad laser membrane does not introduce scattering losses. Additional L–I characteristics of three device architectures—membrane, wing-like, and no-etch LDs—are shown in Figure S6 in the Supporting Information.

Figure 7b presents the high-resolution lasing spectra for the air-clad LD and the reference LD. Multimode lasing is recorded in pulse mode for both devices. The lasing wavelength is 456 nm for the membrane air-clad LD and 454 nm for the reference device. The emission wavelength of all the studied air-clad LDs—membrane and wing-type—is slightly red-shifted with respect to the reference device from the same wafer. There are two factors that could be the cause of the observed shift. First, during the laser pulse, the temperature of the etched LDs might increase due to limited heat dissipation in the air-cladding geometry, leading to a decrease in the bandgap. Second, theoretical calculations incorporating deformation potentials^40^ indicate that strain affects the band structure of the QW, leading to a shift of the gain spectrum toward longer wavelengths upon relaxation. This effect is also expected to occur in our structures, which exhibit a certain degree of elastic relaxation. We have observed that the red-shift increased with the driving current, which suggests that the first factor is the primary one. Further studies are necessary to clarify the influence of strain relaxation on device performance.

### Air-Cladding Architecture for Long-Wavelength LDs

In order to elucidate the role of the laser membrane thickness for efficient light confinement, we modeled the current design of the laser using COMSOL Multiphysics. The refractive indices of the InGaN and AlGaN layers were calculated using the formulas proposed by Laws et al.^41^ The GaN waveguide thickness, *D*, was varied from 5 to 3000 nm. The rest of the epitaxial structure, i.e., the 50 nm GaN, 2 × 110 nm In_0.04_Ga_0.96_N, QW, and EBL, as well as the TJ region, were kept unchanged. Note that the varied GaN waveguide is symmetrical on both sides of the active region; therefore, the total membrane thickness includes twice the GaN waveguide thickness. Additionally, we studied the influence of the air-cladding design on longer-wavelength emitters by varying the In composition in the QW from 18% for blue (455 nm),) 28% for green (520 nm), and 40% for red (630 nm) emitters. Figure 8a,b presents the refractive index profiles and corresponding optical mode profiles for two blue LD designs: the experimentally studied laser structure with *D* = 500 nm and a theoretical structure with *D* = 100 nm. In the current demonstration, the laser membrane is about 1.4 μm thick, and in the second considered case, the thickness is reduced to about 0.6 μm, resulting in confinement factors of 0.0329 and 0.0424, respectively. Thinning the LD structure results in a significant boost of optical mode confinement by a factor of 1.29. Practical realization of such a thin laser membrane would, therefore, result in an improvement of the LD parameters.

**Figure 8:**
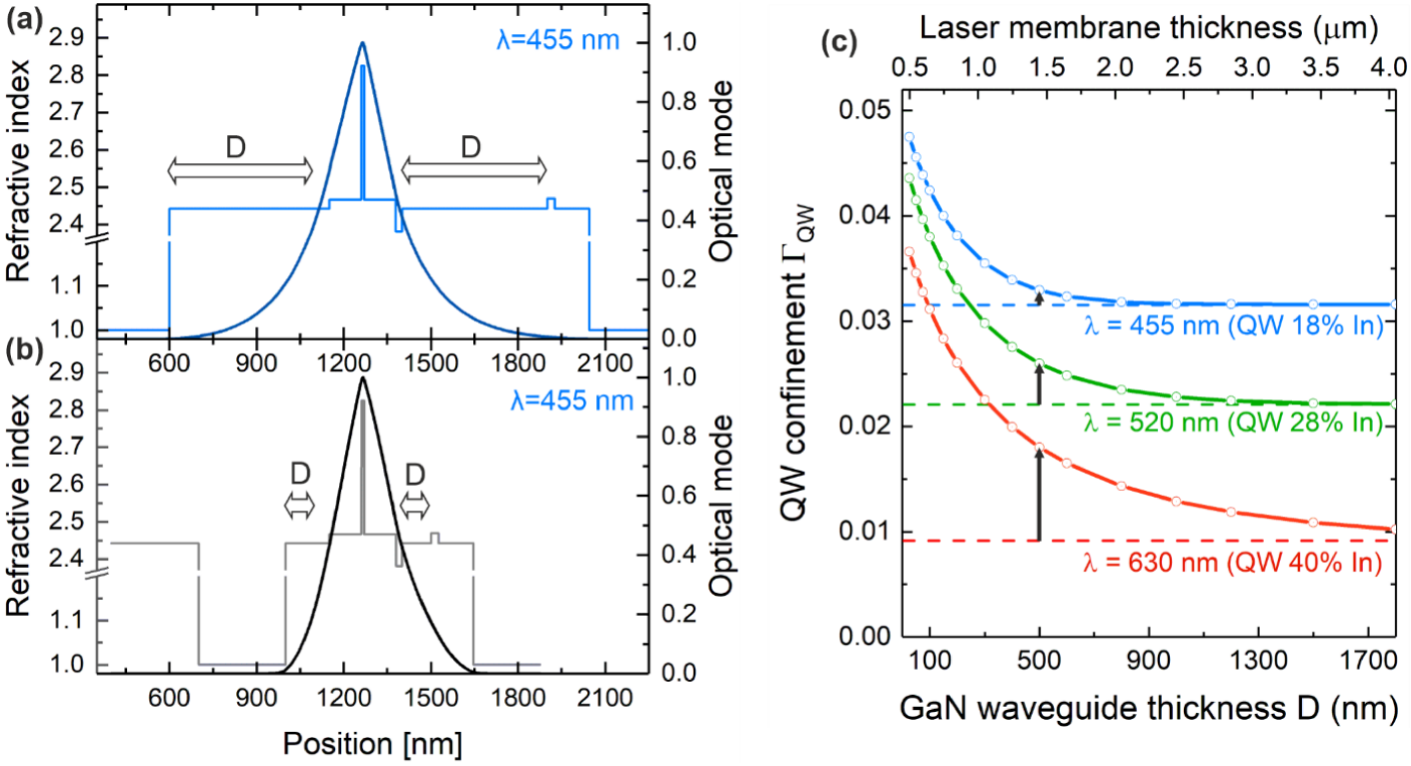
Refractive index profile and optical mode profile for (a) the demonstrated air-cladding LD structure design with GaN waveguide thickness *D* = 500 nm and (b) a thinner laser membrane design with *D* = 100 nm. (c) Calculated confinement factor in the QW for the blue, green, and red air-cladding LDs with varying GaN waveguide thickness, *D*. The emission wavelength is tuned by the In composition in the QW. Dashed lines show the confinement factor for designs without under-etched bottom cladding. Arrows indicate the benefit of the air cladding for fixed *D* = 500 nm for different LDs.

It is well established that light confinement becomes more difficult for longer wavelengths due to smaller refractive index differences between GaN and AlGaN alloys.^42^ Therefore, we consider the implementation of an air cladding to overcome this problem. The blue curve in Figure 8c presents the calculated optical confinement factor in the QW for the fabricated air-cladding lasers as a function of GaN waveguide thickness, *D*, demonstrating that light confinement improves with decreasing membrane thickness. The base level, shown as the dashed blue line, is the reference for the structure without under-etching (GaN bottom waveguide and cladding). For the blue, green, and red LDs, the base levels are 0.0315, 0.0221, and 0.0092, respectively. Interestingly, we note that in the case of the blue LD, the difference between the reference and the demonstrated air-cladding LD is not significant, as indicated by the black arrow. Indeed, this verifies the experimental observation that the parameters of the air-clad membrane lasers and reference LDs are similar. However, with increasing lasing wavelength, the benefit in light confinement of the air-cladding LD structure becomes much more pronounced, as indicated by the black arrows. In the case of blue, green, and red LDs with air cladding, for *D* = 500 nm, the light confinement will be 0.0329, 0.0260, and 0.0180, respectively, thus increasing by a factor of 1.04, 1.18, and 1.96 compared to GaN cladding.

To extend the proposed membrane LD design to longer-wavelength emitters and fully exploit the advantages of the air-cladding architecture, additional challenges must be addressed, particularly regarding the optical and structural quality of the high-indium-content active region. The reduced gain in high-indium-content quantum wells (QWs) can be compensated for by an increased optical confinement factor (Γ). Moreover, due to the higher strain in such structures, a symmetric membrane design with respect to both composition and layer thickness will be required, as demonstrated for the blue LDs discussed in this work.

## Conclusions and Outlook

In this work, we experimentally demonstrate the novel design of nitride-based laser diodes (LDs) incorporating both top and bottom air-claddings. The top air-cladding is realized through a side top contact, which is connected to a tunnel junction via a regrown layer. The bottom air-cladding is formed by an air gap created through electrochemical etching (ECE). Very smooth surfaces are observed by AFM after the removal of the sacrificial layer. The measured surface root-mean-square roughness (rms) at the backside of the laser membrane is 0.66 nm for an area of 5 × 5 μm^2^. Nanobeam SXDM at beamline ID01/ESRF revealed elastic stress relaxation of the whole under-etched, free-standing layer stack, facilitated by the equilibration of lateral strains in the remaining GaN and InGaN layers. This also led to lattice bending and a vertical displacement of the laser of a few nanometers . The developed fabrication route integrates the ECE process after the full epitaxial structure growth. Electrical and optical characterization of the fabricated devices, operated in pulsed mode, shows promising performance, as the threshold current and slope efficiency were comparable to or better than the reference LDs from the same wafer. Further optimization steps should include improvements in the structure design in terms of electrical parameters and reducing the membrane thickness to benefit from the boost in light confinement. Presented results of theoretical modelling confirm air-cladding architecture as a promising solution to the poor light confinement in the long-wavelength (green–yellow–red) emittersproved to be a potential . In fact, the proposed air-cladding architecture could also be a viable alternative for ultraviolet (UV) LDs grown on GaN substrates, for which strain relaxation is a serious issue. Last but not least, the role of ECE as a technique for device liftoff is highlighted, providing excellent selectivity in material removal with control over the etch front advancement. This opens the way to fully transferable devices and substrate reuse.

## Methods

### Epitaxy and Processing

The epitaxial structure was grown by plasma-assisted molecular beam epitaxy (PAMBE) on a (0001) Ammono-GaN substrate in a Veeco Gen20A reactor. As shown in Figure 1a, first, a 100 nm GaN:Si layer with a Si concentration of [Si] = 5 × 10^18^ cm^–3^ was grown. Next, a heavily doped 300 nm In_0.04_Ga_0.96_N:Ge sacrificial layer was grown, which consisted of 260 nm with a doping level of [Ge] = 2 × 10^20^ cm^–3^ and 40 nm with [Ge] = 6 × 10^20^ cm^–3^. The growth finished with a 10 nm undoped In_0.04_Ga_0.94_N layer. Then, the growth continued with a 550 nm GaN:Si layer with [Si] = 5 × 10^18^ cm^–3^, 110 nm In_0.06_Ga_0.94_N, a 10.4 nm In_0.17_Ga_0.83_N quantum well (QW), and 110 nm In_0.06_Ga_0.94_N. Next, an electron blocking layer (EBL) of 20 nm Al_0.14_Ga_0.86_N:Mg was grown, followed by a 500 nm GaN:Mg layer with [Mg] = 5 × 10^18^ cm^–3^. Next, the TJ part was grown which consisted of 20 nm In_0.02_Ga_0.98_N:Mg with [Mg] = 3.6 × 10^19^ cm^–3^, 3 nm In_0.15_Ga_0.85_N:Mg with [Mg] = 4.8 × 10^19^ cm^–3^, 3 nm In_0.15_Ga_0.85_N:Si with [Si] = 4.4 × 10^19^ cm^–3^, 20 nm In_0.02_Ga_0.98_N:Si with [Si] = 3.4 × 10^19^ cm^–3^. Then, a 20 nm thick n-type GaN:Si layer [Si] = 3.5 × 10^19^ cm^–3^ was grown. After the TJ, the growth process was interrupted in order to define laser ridges of 3 μm and 5 μm widths by standard lithography and dry etching to a depth of 70 nm. Finally, a 100 nm GaN:Si layer with [Si] = 3 × 10^19^ cm^–3^ was grown.

In the case of membrane (wing-like) LDs, the fabrication of the openings for ECE consisted first of dry-etching 15 μm × 15 μm windows (15-μm-wide grooves) to a depth of 1150 nm, reaching the middle of the GaN:Si layer, see Figure 1c. Second, a dielectric stack of 100 nm SiO_2_/1000 nm SiN/100 nm SiO_2_ was deposited. Third, photolithography and reactive ion etching (RIE) utilizing SF_6_ were used to open 5 μm × 5 μm windows (5-μm-wide grooves). Lastly, RIE in Ar and Cl mixtures was used to etch an additional 480 nm down to the middle of the sacrificial InGaN:Ge layer, as illustrated in Figure 1c. The total depth of the windows (grooves) for ECE was 1630 nm.

ECE was carried out in 0.3 M oxalic acid at 2.5 V.^18^ The wafer was divided into two pieces before ECE to test the etch rate of the sacrificial layer. The smaller piece was test-etched for 270 min, while the larger piece was etched for 408 min. The etch front was measured by an optical microscope to be 23 μm from the window (groove) edge, as marked with the arrows in Figure 2a-b, which corresponds to an etch rate of 0.056 μm/min. The rate of the test-etch was very similar, 0.050 μm/min, as the etch front in the test piece was 13.5 μm. The protective dielectric stack successfully prevented parasitic etching of the active region and the highly doped TJ. The last part of the processing involved substrate thinning and metal contact deposition. For the top and bottom n-type contacts, the following metal stacks were deposited: top Ti/Al/Ni/Au (30/60/40/75 nm) and bottom Ti/Pt/Au (80/100/270 nm).

### Membrane Surface Characterization

AFM: Surface topography was characterized by *Veeco Instruments Nanoscope 3100* in tapping mode. Data were processed in Gwyddion 2.68.

A SEM: Zeiss Leo 1530 scanning electron microscope with InLens and secondary electron detectors was used to characterize the topography of the LDs after processing.

### Scanning X-ray Diffraction Microscopy

Scanning X-ray diffraction microscopy measurements have been carried out at the ID01 beamline of the European synchrotron (ESRF).^33^ The X-ray energy for the experiment was set to 8.8 keV, and the beam was focused to a spot size of ∼30 nm using a Fresnel Zone Plate. Bragg reflections were aligned in vertical coplanar reflection geometry. For spatial mapping in (*x, y*), the sample was scanned continuously across the beam while the diffracted X-rays were recorded with a *Maxipix* area detector. By repeating the spatial raster scan for a series of beam incidence angles η (rocking scan), a five-dimensional SXDM data set was recorded, containing a three-dimensional (3D) RSM at each (*x, y*) position. A *FalconX* energy-resolved fluorescence detector simultaneously recorded the indium *L-*edge fluorescence for tracking sample drift. During data processing, the positions of the 0006 Bragg peaks for both GaN and InGaN layers were determined in spherical coordinates from each 3D RSM, thus providing spatial maps of the lattice parameter *c* = 6 × d_0006_ = 6 × 2π/|*Q*_*z*_| and the two components of lattice tilt (φ and θ).^32^

## Supplementary Material



## Data Availability

The data supporting the findings of this study, including the plots presented in the main text, are available within the article and its Supporting Information or from the corresponding author upon request. The raw synchrotron data are accessible under doi.org/10.15151/ESRF-ES-1687674103, and derived data are available from the authors upon reasonable request. Additional data can also be downloaded from the open repository https://doi.org/10.18150/J39P0I.

## Notes

SI contains SEM images showing the LD membrane after detachment and the processed wafer from which this membrane was taken (Figure S1), supplementary synchrotron-based scanning X-ray diffraction microscopy (SXDM) data (Figures S3–S5), a collection of light current characteristics for the membrane-LDs, wing-LDs, and reference no-etch LDs measured on another setup (Figure S6), and mobile phone camera images of the far-field pattern for membrane and non-etched LDs (Figure S7) (PDF)
