# Supplementary material for: Air-Cladding Blue Laser Diodes
Source: ACS Appl Mater Interfaces. 2026 Jun 20;18(25):35483–92. doi: 10.1021/acsami.6c01600 (PMC13339020; doi:10.1021/acsami.6c01600)
Supplement: Supplementary file 1 [file am6c01600_si_001.pdf]

Supporting Information

Air-cladding Blue Laser Diodes

Marta Sawicka[1,], Mateusz Hajdel[1], Oliwia Gołyga[1], Henryk Turski[1], Mikołaj Chlipała[1], Anna Feduniewicz[1], Szymon Stańczyk[1], Czesław Skierbiszewski[1], Cedric Corley-Wiciak[2], Carsten Richter[3], and Grzegorz Muziol[1]*

[1] Institute of High Pressure Physics Polish Academy of Sciences, Sokołowska 29/37, 01-142 Warsaw, Poland

[2] European Synchrotron Radiation Facility, X-ray Imaging and Microscopy Group, 71 Avenue des Martyrs, CS 40220, 38043 Grenoble Cedex 9, France

[3] Leibniz -Institut für Kristallzüchtung, Max-Born-Straße 2, D-12489 Berlin, Germany

*Corresponding Author email address: sawicka@unipress.waw.pl

To investigate the backside surface roughness of the air-cladding laser diodes (LDs), a simple method based on adhesion and selective detachment was employed. A piece of conductive carbon tape, commonly used for mounting samples in Scanning Electron Microscopy (SEM) studies, was applied to a piece of the processed wafer that had not yet been cleaved into individual devices. Upon subsequent removal of the tape, those portions of the under-etched membrane that adhered sufficiently strongly, were detached from the wafer. This process involved no mechanical scratching.

Figure S1 presents SEM images acquired from (a) the top view of the GaN wafer after the “scotch-tape” detachment of the LD membrane, and (b) the backside surface of the detached LD membrane, with its top surface remaining attached to the carbon tape. The locations selected for Atomic Force Microscopy (AFM) measurements are indicated. Figure S1(c) and Figures S1(d) provide higher-magnification views of the same regions. The surface looks very smooth and minor traces of residual debris can be observed.

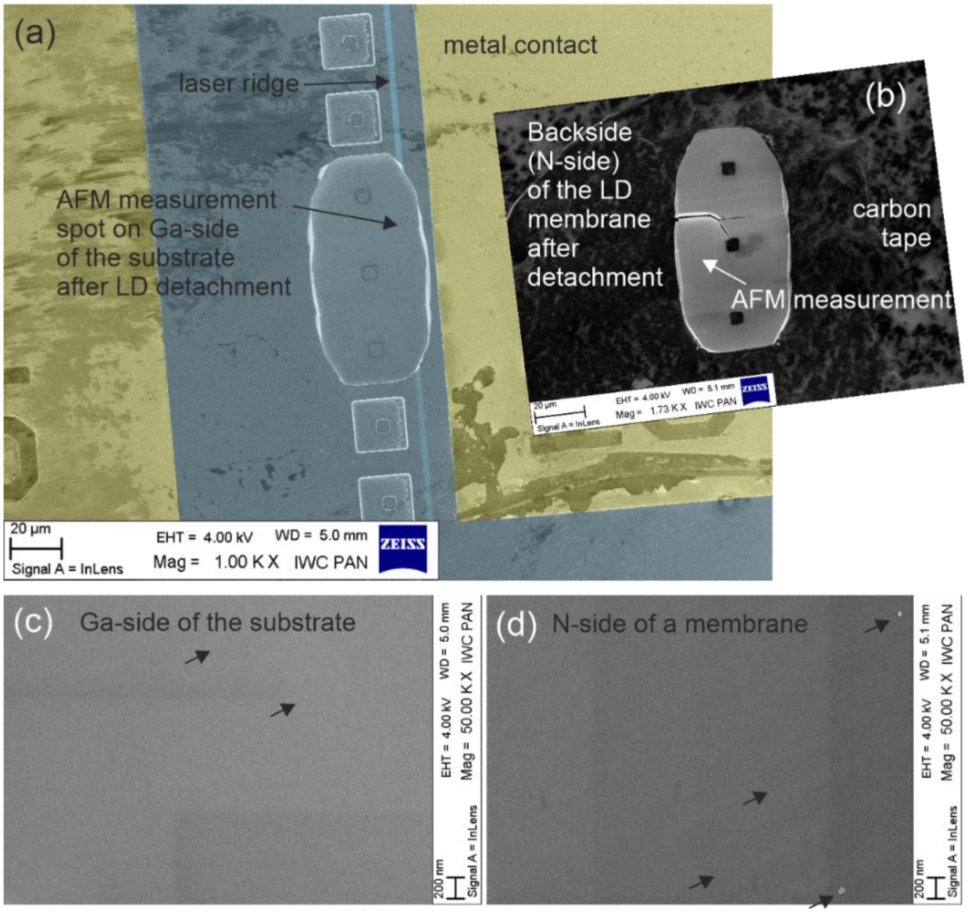

Figure S1. SEM images showing (a) piece of the wafer with processed lasers (false color shows the positions of the metallization and laser ridge) after under-etched membrane detachment, (b) the detached piece of the LD membrane on a carbon tape with annotated spot of the AFM measurement. Respective larger magnification images of the (c) Ga-side of the substrate after LD detachment and (d) N-side of the LD membrane. Short arrows indicate particles visible.

Data collected by synchrotron-based scanning X-ray diffraction microscopy (SXSM) for the under-etched membranes and wing-type lasers and discussed in the main text of the article are complemented by the maps of X-ray diffraction intensity for membrane-type and wing-type air-clad LDs (Figure S2 left and right, respectively) and calculated reciprocal space maps in specific location of the measured maps, allowing for the comparison of strain state in the area above the air-gap with the reference region with the sacrificial layer for the membrane-type and wing-type air-clad LDs, shown in Figure S3a and S3b respectively. Figures S4 and S5 show extracted cuts from the RSMs supporting the analysis of the strain state in the membrane- and wing-type structures in reference to the regions that are not etched.

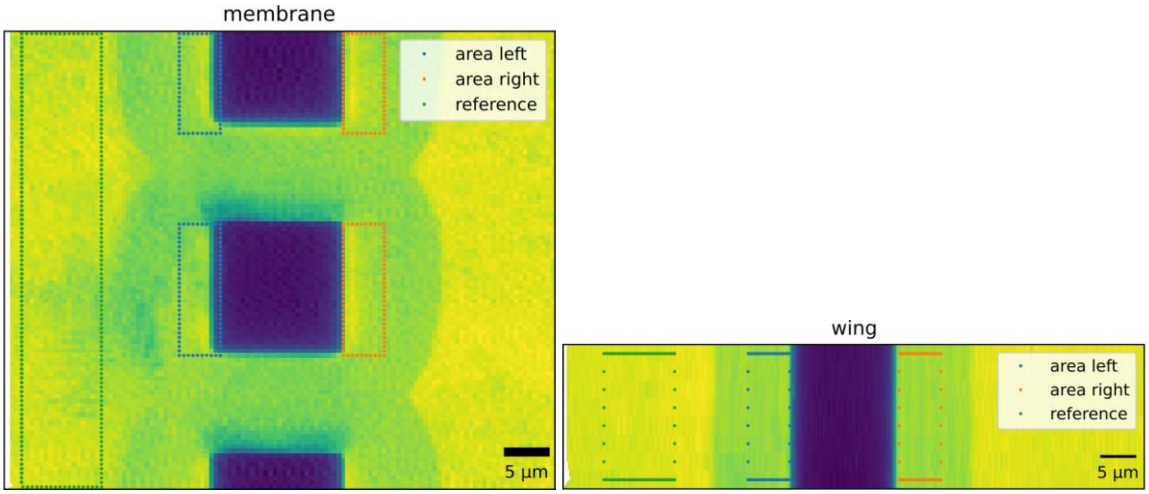

Figure S2: Maps of SXDM diffraction intensity for the etched membrane (left) and wing (right) structures. Areas for which reciprocal space maps (RSMs, see Figure S3) were computed are marked by surrounding dots.

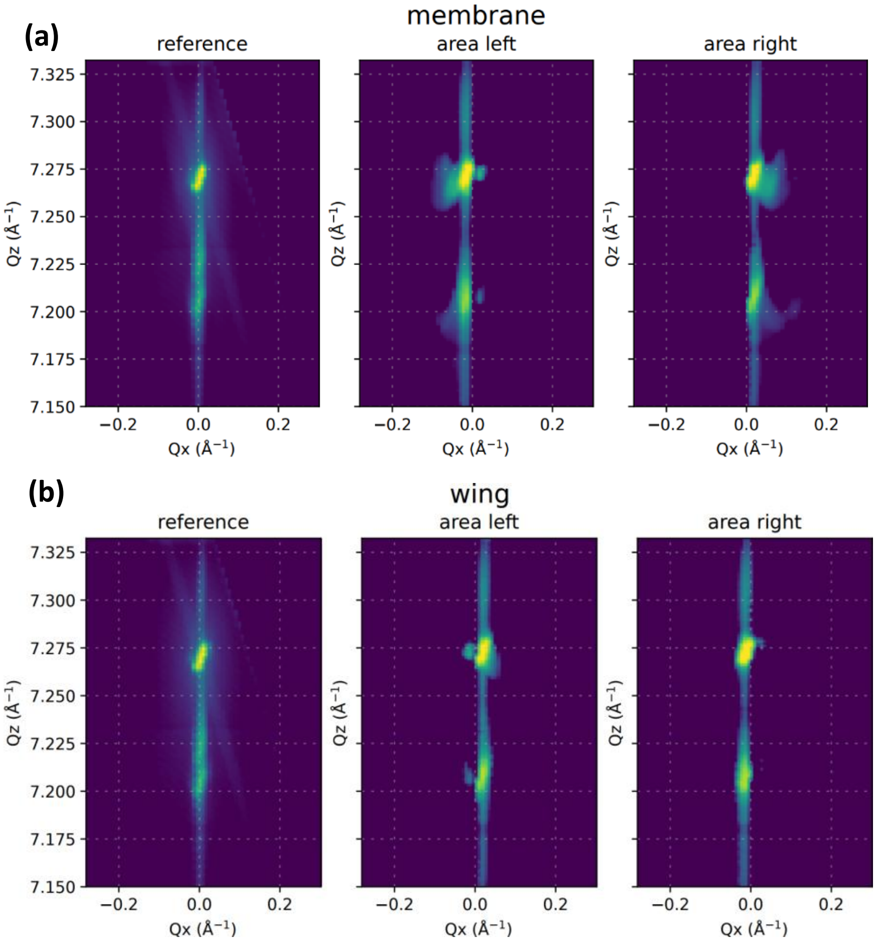

Figure S3: 2D RSMs extracted for the regions in the under-etched (a) "membrane" structure marked in Figure S2(left) and (b) „wing" structure marked in Figure S2(right) . In both cases, the reference RSM has been subtracted from RMSs for "area left" and "area right".

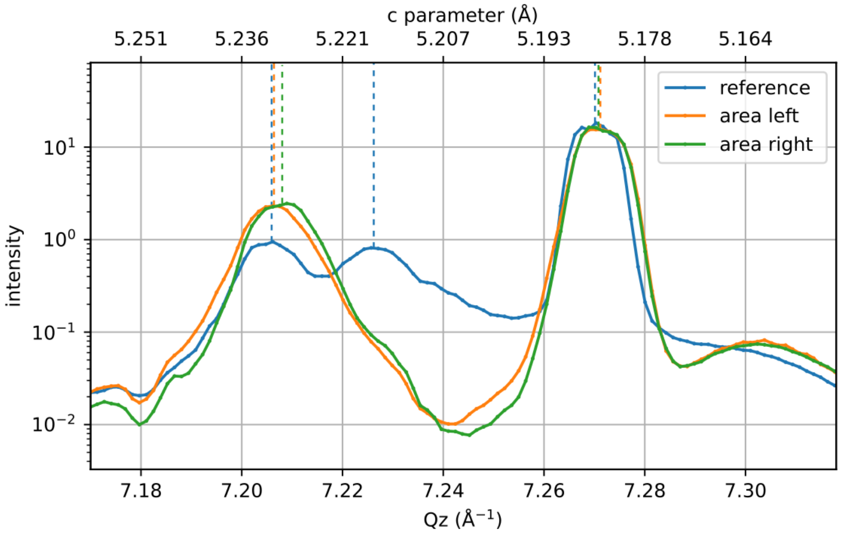

Figure S4: 1D RSMs (radial cut) extracted from the 2D RSMs of the "membrane" structure in Figure S3(a) by integration along Q_x in the interval [-0.05 Å^{-1}, 0.05 Å^{-1}]. The extracted cuts peak at the values as marked by vertical dashed lines: reference: 5.2317 Å, 5.217 Å, 5.1855 Å; area left: 5.2313 Å, 5.1847 Å and area right: 5.2302 Å, 5.1850 Å.

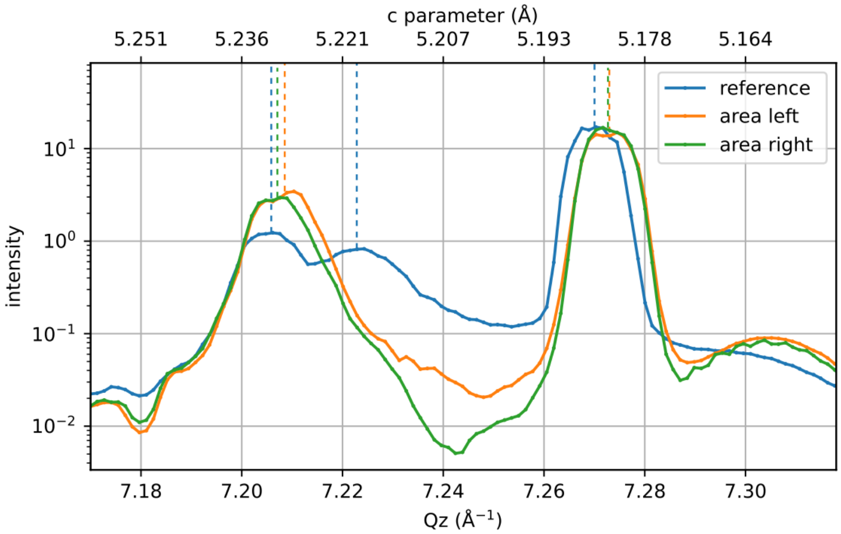

Figure S5: 1D RSMs (radial cut) extracted from the 2D RSMs of the "wing" structure in Figure S3(b) by integration along Q_x in the interval [-0.05 Å, 0.05 Å]. The extracted cuts peak at the values as marked by vertical dashed lines: reference: 5.2317 Å, 5.2194 Å, 5.1856 Å; area left: 5.2298 Å, 5.1835 Å and area right: 5.2308 Å, 5.1837 Å

Electrical measurements of the membrane air-clad and no-etch LDs presented in the manuscript were carried out in an experimental setup equipped with a high-resolution spectrometer. Before these measurements, larger number of devices were characterized on another setup. The collection of Light -Current (L-I) characteristics of the nominally identical device architectures are shown below. The devices presented in the paper were selected as representative based on a criterion of a low threshold current and the data is bolded.

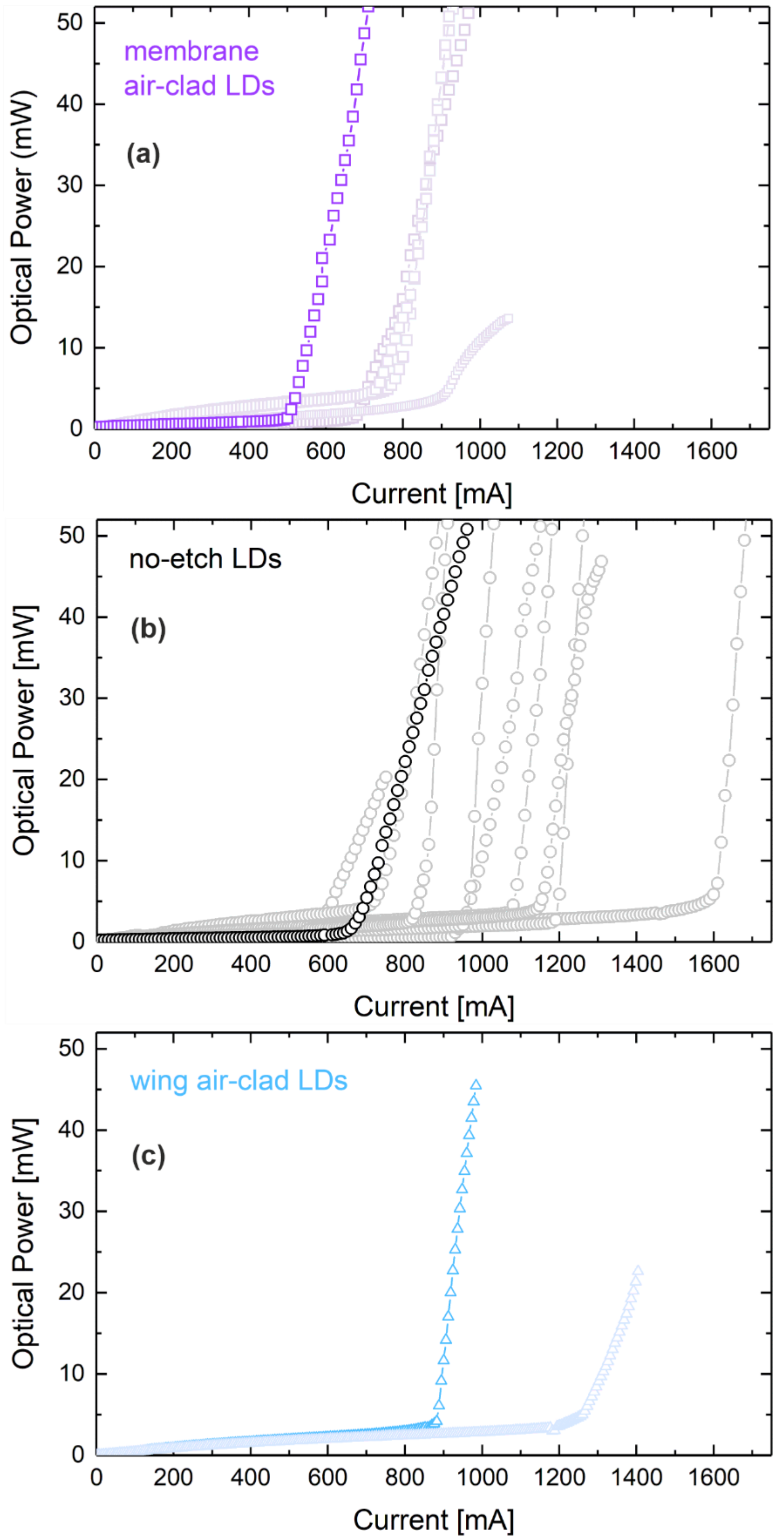

Figure S6. Collection of L-I characteristics for (a) membrane air-clad LDs, (b) no-etch LDs and (c) wing LDs that operated in pulse mode.

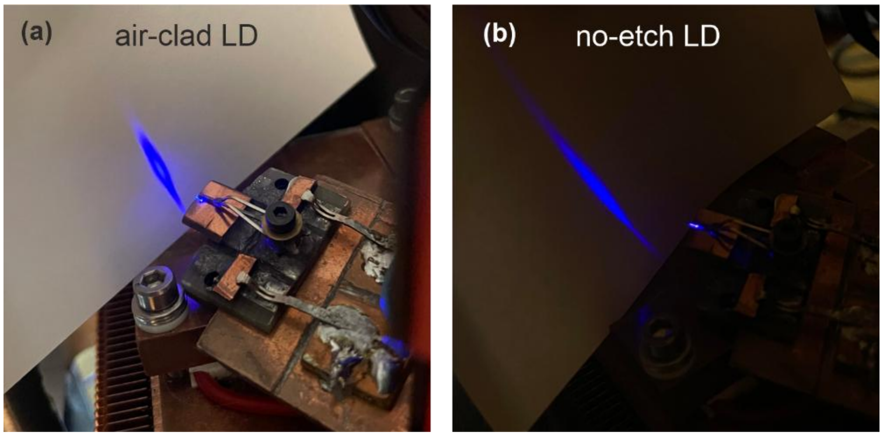

Figure S7: Illustrative photos taken by mobile-phone camera during electrical measurements of the devices, showing the far-field patterns for (a) air-clad LD and (b) reference LD (not etched), that indirectly indicate a similar light confinement in both architectures.

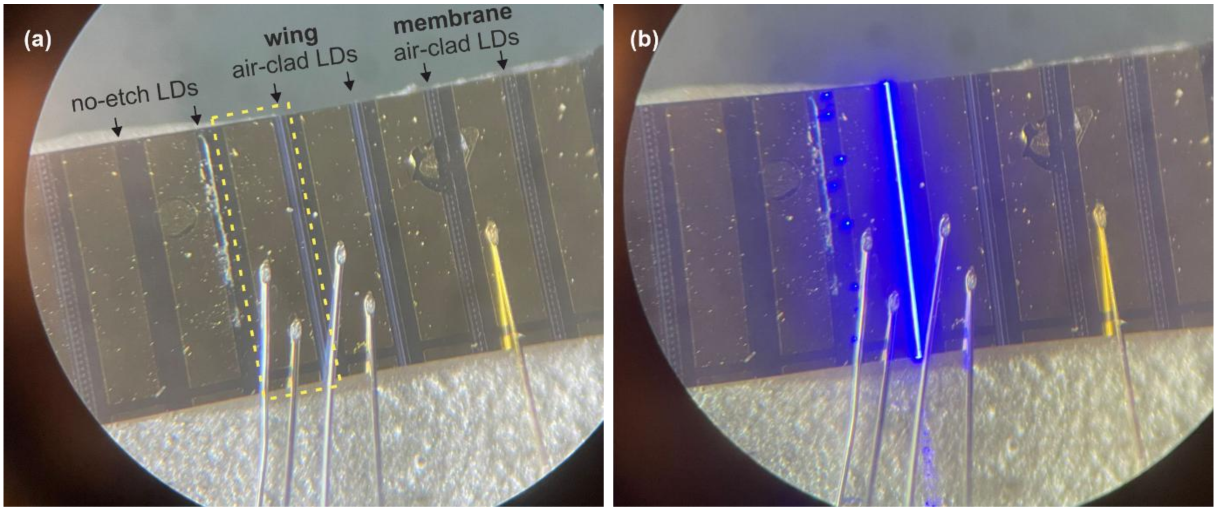

Figure S8: Photos taken by mobile-phone camera through the optical microscope objective during electrical measurements of the devices on a bar with processed lasers. (a) without current injection. Laser ridge positions are marked with arrows. Specific LD architectures: no-etch, wing air-clad and membrane air-clad, respectively are indicated. Single wing air-clad LD device is marked with a dashed rectangle. (b) Photo of the same laser bar taken during wing air-clad LD operation under pulse mode above threshold at 850 mA.
